# Triple Vessel Coronary Artery Disease Identified in a 21-Year-Old Female

**DOI:** 10.7759/cureus.48975

**Published:** 2023-11-17

**Authors:** Yashitha Chirumamilla, Srujan Edupuganti, Luay Alkotob

**Affiliations:** 1 Internal Medicine, Hurley Medical Center, Flint, USA; 2 Internal Medicine/Pediatrics, Hurley Medical Center, Flint, USA; 3 Cardiology, Hurley Medical Center, Flint, USA

**Keywords:** lad stenosis, congestive heart failure, dyslipidemia, triple vessel disease, atherosclerosis, coronary artery disease

## Abstract

Premature coronary artery disease (CAD) is characterized by the presence of symptomatic atherosclerosis in the coronary circulation in males below the age of 55 and females below the age of 45. We present the case of a 21-year-old female with a past medical history of heart failure with preserved ejection fraction, poorly controlled diabetes mellitus, essential hypertension, nephrotic syndrome, dyslipidemia, and class I obesity who presented with complaints of worsening bilateral lower extremity edema and exertional shortness of breath. Given her physical examination findings and laboratory investigations, a diagnosis of heart failure exacerbation was made. Echocardiography revealed a significant change in ejection fraction from three months earlier, and thus, she underwent a nuclear stress test. She was found to have fixed perfusion defects in the inferior wall. A diagnostic left heart catheterization identified severe triple vessel disease affecting the left anterior descending, left circumflex, and right coronary arteries. The patient and her family opted against coronary artery bypass grafting, and she was discharged to pursue high-risk intervention as an outpatient. This case highlights the importance of strict regulation of modifiable risk factors for CAD even in teenagers and young adults as her disease process likely began several years prior to the ultimate development of triple vessel CAD.

## Introduction

Premature coronary artery disease (CAD) can be defined as symptomatic coronary artery obstruction due to atherosclerotic lesions with onset prior to the age of 45 in males and 55 in females [1]. The development of CAD has been studied extensively in middle-aged and elderly individuals, but the disease process has largely been overlooked in younger adults. The disease process of atherosclerosis begins at a young age, but the symptomatic incidence of CAD in adults less than 40 years of age accounts for about 3% of total cases according to studies [2]. We present the case of a 21-year-old female presenting with triple vessel CAD.

## Case presentation

**Figure 1:**
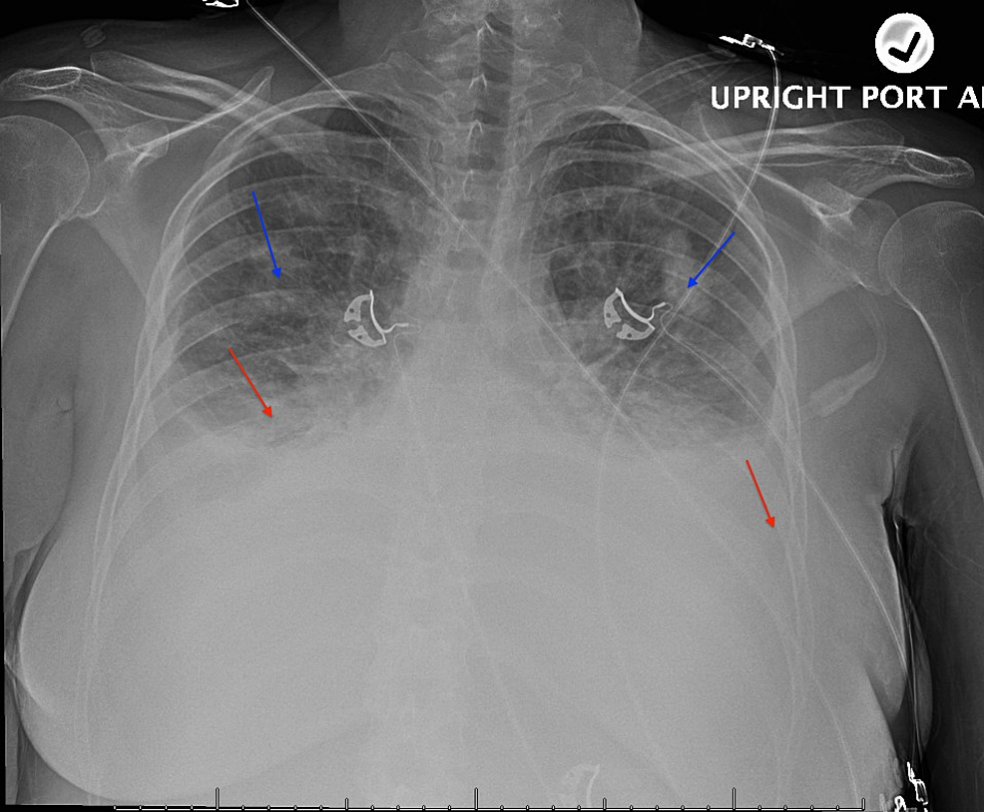
Bilateral pleural effusions with blunting of the costophrenic angles associated with interstitial edema as indicated by the red and blue arrows, respectively

A 21-year-old Caucasian female with a past medical history of heart failure with preserved ejection fraction, poorly controlled type 1 diabetes mellitus, essential hypertension, nephrotic syndrome, dyslipidemia, and class I obesity (BMI 33.1) presented to the emergency department with complaints of lower extremity swelling associated with weight gain over the past week along with exertional shortness of breath. Her vital signs on presentation revealed a blood pressure of 168/118, heart rate of 110, and respiratory rate of 20. She was requiring 4 L of oxygen via nasal cannula to maintain appropriate oxygen saturation. On physical examination, she was found to have bilateral lower extremity pitting edema up to the level of the thighs, jugular venous distension, and diminished lung sounds bilaterally at the bases. Laboratory evaluation was significant for a mildly elevated troponin of 0.043 ng/mL (reference range: <0.040 ng/mL) and a brain natriuretic peptide value of 1499 pg/mL (reference range: <100 pg/mL). Her lipid panel revealed a normal total cholesterol and low-density lipoprotein level with a low high-density lipoprotein level of 22 mg/dL (reference range: 40-59 mg/dL). Her electrocardiogram (EKG) showed no acute ischemic changes. Her chest radiography revealed pulmonary edema with bilateral pleural effusions and cardiomegaly (Figure 1). 

**Figure 2:**
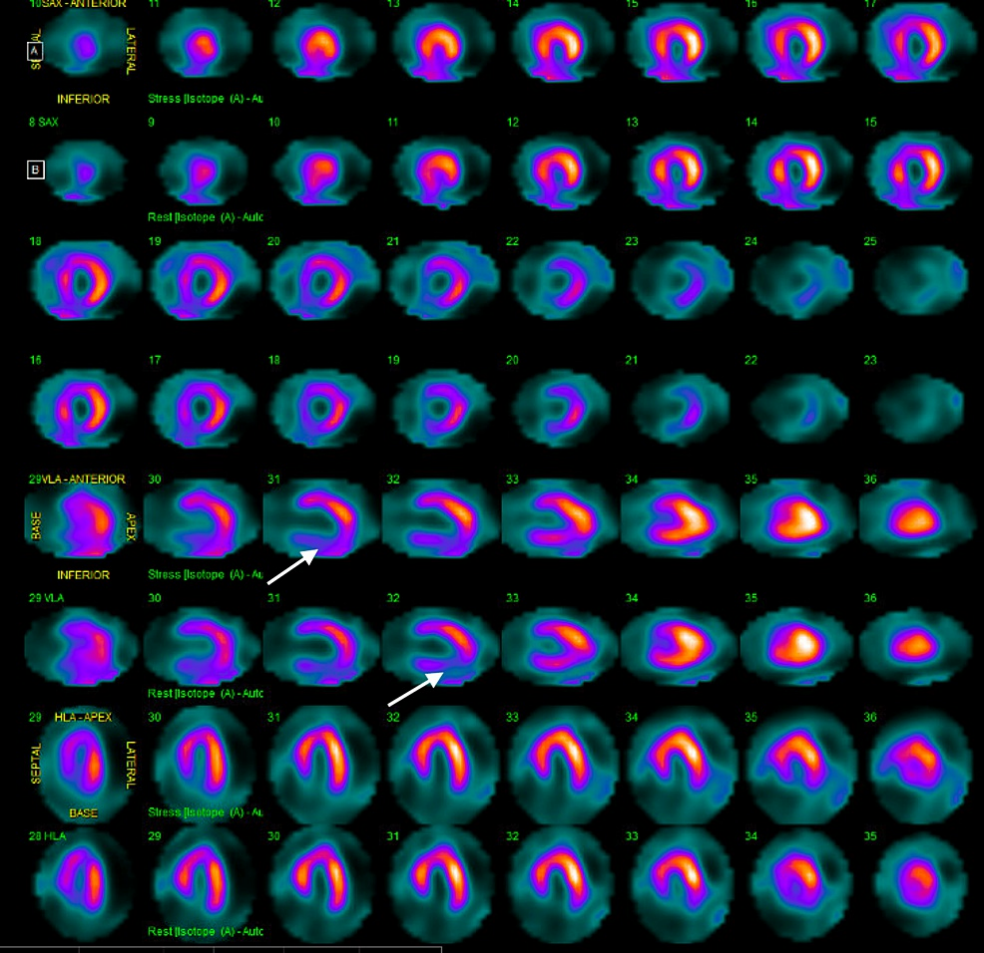
Fixed perfusion defects at the inferior wall as indicated by the arrows

She was diuresed with intravenous furosemide, and Cardiology was consulted for the further management of her congestive heart failure. A repeat echocardiogram performed during this admission identified a reduction in her ejection fraction from 55-60% to 40-45% with global hypokinesis. One week prior to this presentation, the patient underwent a cardiac MRI as an outpatient that showed delayed enhancement in the subendocardial inferior wall and a reduced ejection fraction which would signify an old right coronary artery infarct which was assumed to be highly unlikely for her age. Her significant change in ejection fraction in a period of three months on both cardiac MRI and echocardiography demanded further ischemic workup to be done in the form of a nuclear stress test. The stress test revealed fixed perfusion defects in the inferior wall (Figure 2).

**Figure 3:**
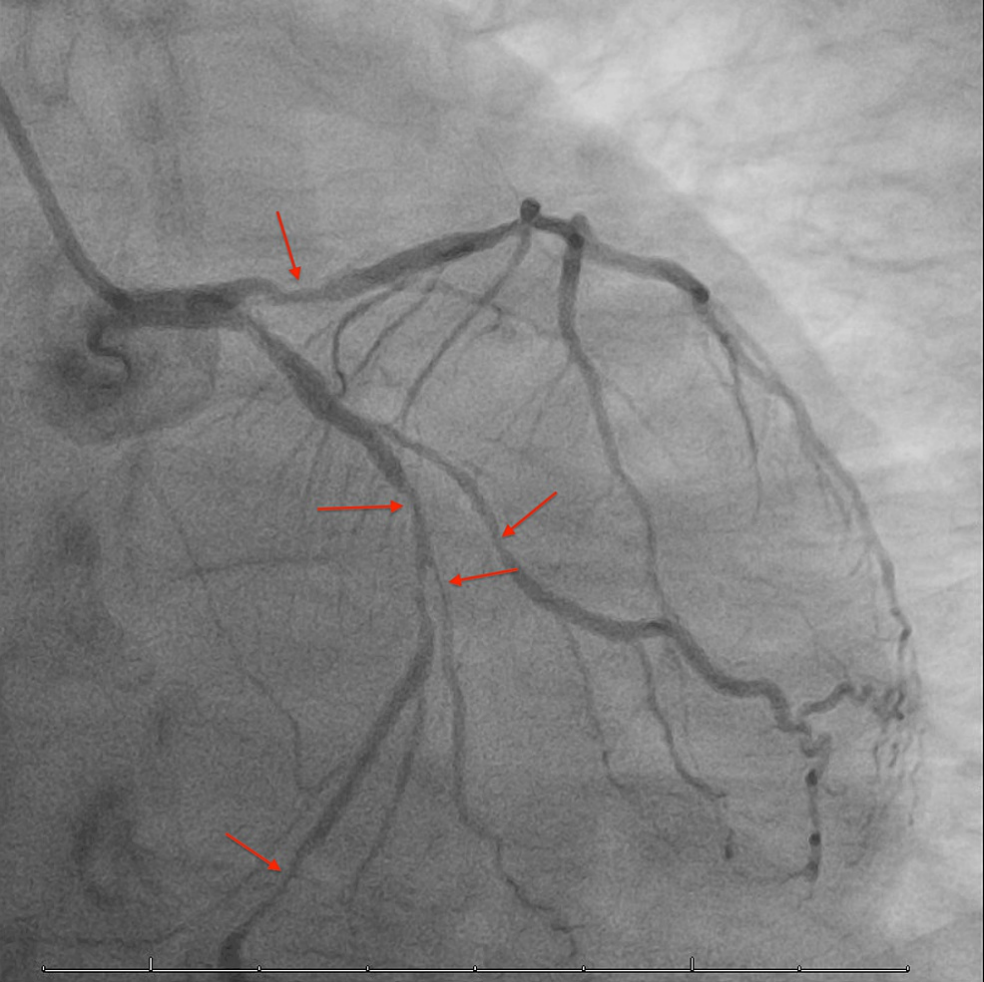
Multifocal occlusions in the left anterior descending and left circumflex arteries including their branches as indicated by the arrows

**Figure 4:**
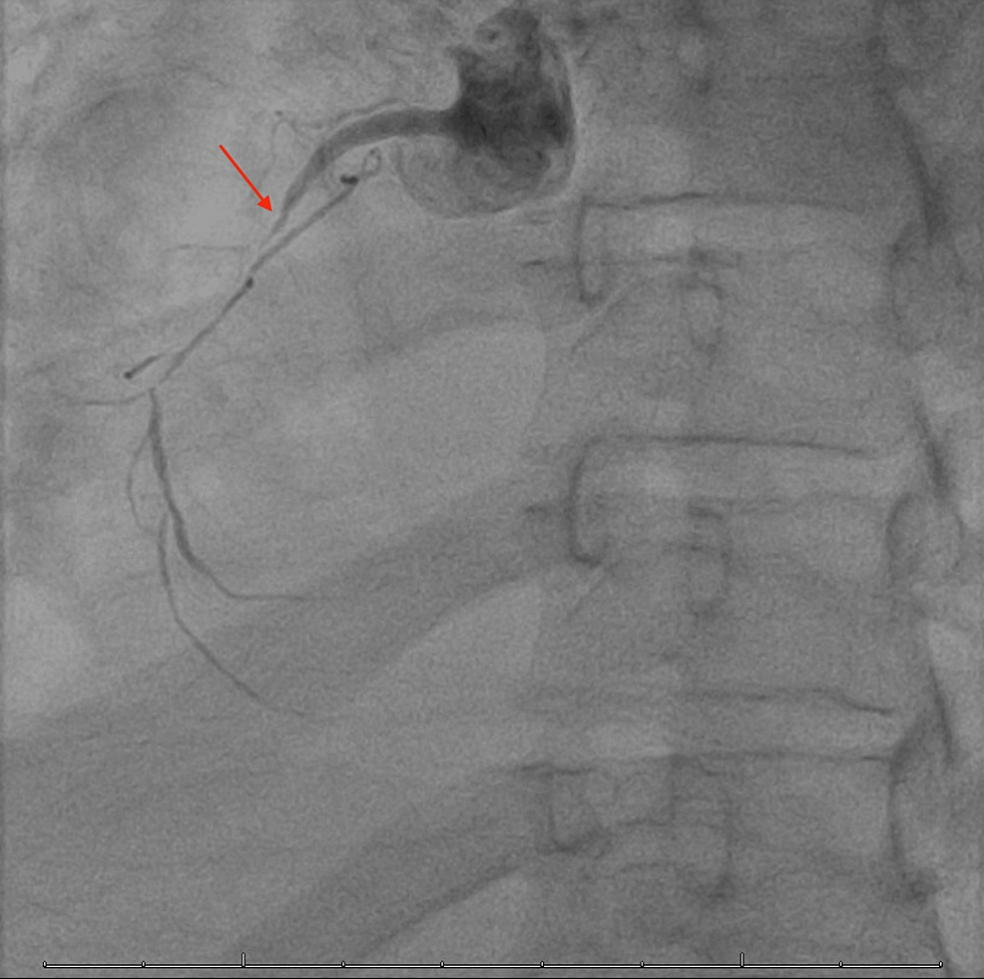
Total occlusion of the proximal right coronary artery as indicated by the arrow

For further diagnostic purposes, she had a left heart catheterization which showed 60% stenosis of the proximal, 80% of the mid, and 90% of the distal left circumflex artery along with 70-90% stenosis of the left anterior descending artery and total occlusion of the proximal right coronary artery with faint collaterals (Figure 3 and Figure 4).

The patient and her family refused the option of coronary artery bypass grafting, and she was ultimately discharged wishing to follow up outpatient for a high-risk intervention after the optimization of her guideline-directed medical therapy with dual antiplatelet therapy, beta-blocker, statin, and vasodilator. 

## Discussion

The true prevalence of premature CAD is unknown because most young individuals remain asymptomatic and do not undergo medical interventions. An autopsy study of 760 victims of accidents, suicides, and homicides aged 15-34 revealed advanced coronary atherosclerotic plaques in only 2% of males aged 15-19 and none of the females. Advanced disease was identified in 20% of males and 8% of females between the ages of 30 and 34 [3]. Our patient presented with symptomatic triple vessel CAD at the age of 21 signifying the onset of atherosclerosis likely in her early teenage years.

The pathophysiology of atherosclerosis can be summarized as increased plasma cholesterol levels causing changes in arterial endothelial permeability, allowing lipids into the artery wall. Circulating monocytes migrate into subendothelial spaces by the process of diapedesis and eventually become foamy macrophages after engulfing the lipids leading to massive accumulation of intracellular cholesterol. The end result is a cascade of vascular malformations such as intimal thickening, development of fatty streaks, fibroatheromas, and vulnerable plaques [4].

Overall, the same risk factors for atherosclerosis are present among affected older adults and younger adults. Risk factors can be divided into nonmodifiable and modifiable. Nonmodifiable risk factors such as age, gender, ethnicity, and family history of CAD are all absent in our patient. Modifiable risk factors that are most prevalent include tobacco smoking, diabetes mellitus, hypercholesterolemia, hypertriglyceridemia, and low levels of high-density lipoprotein. Early onset of juvenile diabetes mellitus such as in our patient has also been associated with a higher risk of premature CAD. Obesity also has a high correlation with the development of atherosclerosis. It is also crucial to keep in mind uncommon risk factors such as coagulopathies and connective tissue diseases in young patients [2]. Chronic kidney disease contributes to CAD as well by contributing additional risk factors of inflammation and oxidative stress. A systematic review has shown that with a glomerular filtration rate decline below 60-75 mL/min, the risk of developing CAD increases linearly [5].

## Conclusions

Despite the presence of several modifiable risk factors in a poorly controlled fashion, the development of triple vessel disease in a young female is a rare occurrence. It is crucial for physicians to be vigilant regarding the presence of ischemic heart disease in young patients and, when suspected, ensure that further diagnostic and therapeutic interventions are done.

Reinforcing strict regulation of modifiable risk factors for atherosclerosis such as diabetes mellitus, hypertension, and dyslipidemia is crucial in teenagers similar to older adults. The development of CAD can begin at a young age and become symptomatic prematurely as well such as in our patient. Hence, further major studies regarding screening for CAD in young adults and pediatric patients, especially those with a higher number of risk factors, are necessary for better preventive medicine. Despite a young age, a thorough ischemic workup is also warranted when there are significant changes in cardiac function.
